# Numerous Asymptomatic Brain Tuberculomas Complicated by Fatal Tuberculous Meningitis

**DOI:** 10.7759/cureus.63090

**Published:** 2024-06-25

**Authors:** Irakli Alavidze, Mariam Shubitidze, Gvantsa Khodeli, Shorena Dvali, Aleksandre Tskitishvili

**Affiliations:** 1 Aieti Medical School, David Tvildiani Medical University, Tbilisi, GEO; 2 School of Medicine, Ilia State University, Tbilisi, GEO; 3 HIV/AIDS Department, Infectious Diseases, AIDS and Clinical Immunology Research Center, Tbilisi, GEO

**Keywords:** global health policy, public health, tuberculous meningitis (tbm), medication noncompliance, immunosuppresion, georgia, hiv aids, central nervous system tuberculosis, intracranial tuberculoma

## Abstract

Tuberculosis (TB) is still one of the most challenging infectious diseases worldwide. Coinfection with HIV increases the likelihood of extrapulmonary involvement, including the tuberculosis of the central nervous system (CNS-TB). CNS-TB often presents as tuberculomas or tuberculous meningitis. Although tuberculomas can be single or multiple, asymptomatic carriage of numerous tuberculomas is seldom reported. We present a case of a 55-year-old man who carried at least 34 tuberculomas of different sizes asymptomatically before developing and succumbing to tuberculous meningitis. Furthermore, we highlight several possible public health challenges that might have complicated his clinical course, suggesting that future studies also focus on these variables alongside more traditional clinical issues.

## Introduction

*Mycobacterium tuberculosis* is the causative agent behind tuberculosis (TB). The World Health Organization (WHO) reported 10.6 million new active TB cases developed in 2022, with an incidence rate of 133 per 100,000 [1]. HIV-induced immunosuppression dramatically increases the susceptibility to TB. Adult HIV-positive patients not on antiretroviral therapy show an exponential increase in TB risk with falling CD4^+^ T cell counts [2].

While TB mainly affects the lungs, immunocompromised individuals are at higher risk of extrapulmonary tuberculosis. Central nervous system TB (CNS-TB) is diagnosed in approximately 1% of all TB patients (likely an underestimation given the difficulties of diagnosing and suboptimal reporting of CNS-TB), with HIV-positive individuals' risk being five times higher [3]. It can manifest in various ways, including tuberculomas and tuberculous meningitis (TBM).

Tuberculomas may or may not cause symptoms, but entirely asymptomatic carriage of numerous tuberculomas is rarely reported. This scarcity is why we report the case of a 55-year-old male patient who carried at least 34 tuberculomas of various sizes for months without any symptoms before developing fatal TBM. We also highlight several public health and policy issues around tuberculous meningitis that are worth researching further.

## Case presentation

A 55-year-old man was brought by ambulance to an infectious department in Tbilisi, Georgia, with increasing generalized fatigue, headache, abdominal discomfort, nausea, vomiting, and diarrhea for the past five days. He was diagnosed with HIV in 2023 in Ukraine and has been on antiretroviral therapy inconsistently since then, stopping the medication upon feeling asymptomatic. Later that year, he was diagnosed with toxoplasmic meningoencephalitis and treated with trimethoprim/sulfamethoxazole (TMP/SMX). However, the patient did not adhere to the suppressive TMP/SMX therapy (dosage not recalled by the patient or the caregiver) and developed recurrent symptoms upon returning to Georgia. He was hospitalized and treated with TMP/SMX again (dosage not available) to the resolution of the acute phase. After the discharge, he again did not adhere to the suppressive treatment.

The patient had a history of engaging in unprotected sexual activities with multiple partners. He also had a 40-pack-year smoking burden, drank alcohol occasionally, and denied using any illicit medications. There were no known allergies.

The physical examination revealed vital signs within normal range, including blood pressure of 115/70 mmHg, heart rate of 96 beats per minute with a regular rhythm, respiration rate of 20 breaths per minute, oxygen saturation at 96%, and temperature of 36.8°C. While the patient was not in acute distress, his speech was incoherent, and he was markedly forgetful of current events. His pupils were round and equal, and he could maintain his gaze, but there was evidence of bilateral nystagmus and right-sided esotropia. The neck muscles were rigid, accompanied by flexure of knees and hips upon flexing the neck (Brudzinski sign). Babinski's sign was also positive bilaterally, with the big toes extending upward upon the stimulation of the soles. The skin turgor was low. Weakened vesicular sounds were heard during auscultation, accompanied by muffled sounds on percussion. He had a persistent dry cough.

The patient's CD4^+^ T-cell count was found to be 89 cells/mm^3^. As he exhibited classic signs of meningitis, empirical therapy with ceftriaxone (2 g given intravenously every 12 hours) was started. Intramuscular metoclopramide (2 mg) was administered to help with nausea and vomiting. An antiretroviral regimen of dolutegravir, lamivudine, and tenofovir (Acriptega 50 mg/300 mg/300 mg, one tablet once a day) was begun. Furthermore, pneumocystis pneumonia (PCP) prevention was initiated with TMP/SMX (seven 480 mg tablets daily, divided into three doses (3+2+2)). 

**Figure 1:**
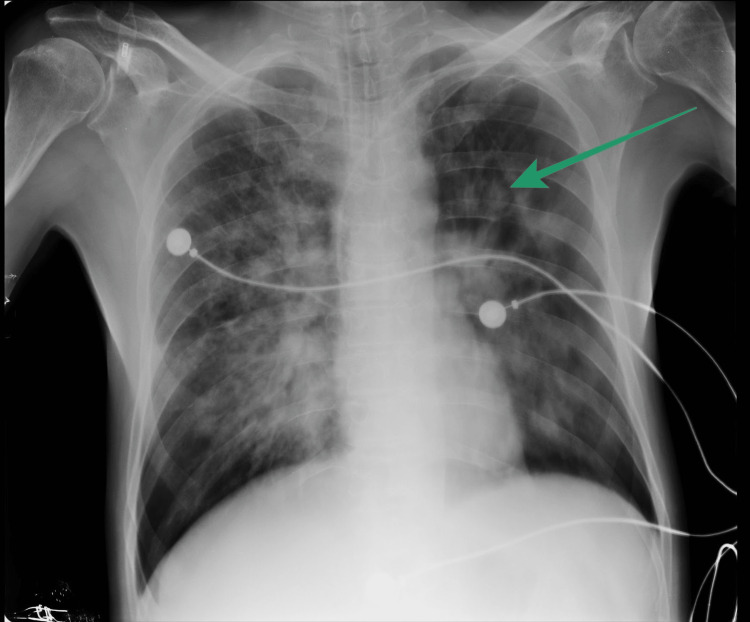
A plain radiograph of the chest suggestive of tuberculous cavitation (anteroposterior view).

Parallelly, given the patient's recent history and subacute presentation, the differential diagnosis of his meningeal symptoms centered on the recurrence of cerebral toxoplasmosis and CNS-TB. A subsequent chest X-ray (Figure 1) revealed cavitary lesions in the left upper lobe, strongly suggesting a mycobacterial infection.

A lumbar puncture was performed to collect cerebrospinal fluid (CSF) for analysis, and the results are given in Table 1. Additionally, nucleic acid amplification by GeneXpert MTB/RIF (Cepheid, Sunnyvale, California, United States) detected *Mycobacterium tuberculosis* without rifampicin resistance.

**Table 1:** Results of cerebrospinal fluid analysis

CSF Analysis	Results	Reference Value
Color	Clear	Clear
White Blood Cell (WBC) Count	66 cells/mm^3^	0–8 cells/mm^3^
Neutrophils	63%	2% ± 5
Lymphocytes	28%	62% ± 34
Monocytes	9%	36% ± 20
Eosinophils	None	None
Protein	1 g/L	0.18-0.45 g/L
Glucose	1.44 mmol/L	2.5–4.4 mmol/L
Microbial Examination	Acid-fast bacilli	Negative

**Figure 2:**
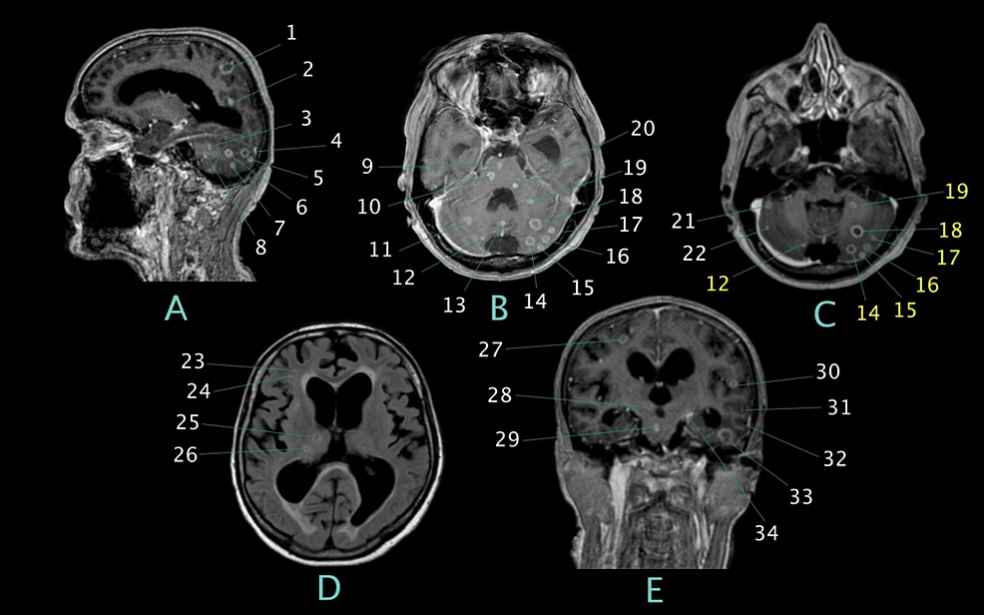
Contrast-enhanced T1-weighted MRI showing 34 tuberculomas. Sagittal (A), axial (B-D), and coronal (E) planes. Due to their number, the lesions are indicated by thin lines. Numbers in yellow indicate duplicate lesions. Unlabeled abnormalities include ventricular dilation due to communicating hydrocephalus, periventricular leukoencephalopathy, and cortical atrophy.

Brain MRI (Figure 2) showed numerous focal intraaxial lesions of different maturation stages above and below the tentorium cerebelli. Some lesions were smaller and nodular, while others exhibited hyperintense ring enhancement and central hypointensity on T1-weighted imaging with contrast. Other significant lesions found on MRI included communicating hydrocephalus, dilation of the large occipital cistern, chronic ventricular hypoxic leukoencephalopathy, and cortical atrophy of the frontotemporoparietal convexity.

On the other hand, polymerase chain reaction testing of blood for *Toxoplasma gondii* was negative. Due to the patient's condition and radiological findings, a biopsy from damaged areas was deemed impossible. Hence, the ring-enhancing lesions in the patient's brain were clinically concluded to be tuberculomas.

Considering the results of imaging and laboratory tests, the empiric antibiotics were discontinued, and the antimycobacterial therapy commenced with rifampicin, isoniazid (INH), and pyrazinamide. Antiretroviral medications were also stopped to prevent further brain damage due to immune reconstitution inflammatory syndrome. However, as the patient's condition kept declining due to a lack of medication response, the patient was transferred to a dedicated TB facility to continue the treatment, where moxifloxacin (400 mg tablet once a day) and linezolid (600 mg tablet once a day) were added to his regimen. His liver function tests exhibited a dramatic increase, necessitating the switch of his medication regimen to amikacin, ethambutol, and linezolid. The patient's status still deteriorated, and since the dedicated TB facility does not maintain an intensive care unit (ICU), he was readmitted to the original hospital in critical condition one month after the previous referral.

Upon readmitting, the patient exhibited signs of shock and end-organ dysfunction. He was in a stupor with a Glasgow Coma Score of 7, exhibiting hypotension, bradycardia, tachypnea, and severe hypoxia. By clinical and laboratory tests, he was diagnosed with acute liver failure, *Klebsiella pneumoniae* pneumonia, and classic AIDS complication of oral thrush. The CD4^+^ T-cell count had fallen down to 18 cells/mm^3^. Within one week, despite further antibiotics (single doses of oral azithromycin 500 mg, intravenous colistin 6 million international units, and intravenous meropenem 2 g), antivirals (two oral 450 mg valganciclovir tablets, twice a day for possible, unconfirmed cytomegalovirus reactivation), and supportive treatment (inotropes, circulatory support), the patient became comatose and expired before further tests were done. Among multiple ongoing disorders, CNS-TB was deemed the primary cause of death.

## Discussion

Brain tuberculomas are nodular space-occupying lesions arising from small metastatic, necrotic tubercles that enlarge or coalesce within the brain parenchyma and can be single or multiple [4]. On this patient's MRI, we have counted 34 tuberculomas. Although disseminated, multiple tuberculomas can be found in the literature, the studies rarely present the actual number of lesions, mostly opting for general (such as "multiple" or "disseminated") or open-ended (>10, >100, etc.) descriptions. Among the articles we found, the highest specific number of tuberculomas in a single brain was 136 [5].

While enlargement of parenchymal tubercles results in tuberculomas, tubercles that occur within or near the meninges (cortical and subcortical spaces), called Rich foci [6,7], may rupture into the arachnoid matter and give rise to TBM. The patient first presented to the hospital when neurological symptoms were very recent (five days). Tuberculomas were in different stages of maturation, and the pattern of CSF cellularity (high neutrophils, relatively low protein count) suggested the early course of TB meningitis [8]. These details point to the extended timeline of tuberculoma presence in the patient's brain before a Rich focus ruptured into the arachnoid space. Thus, it leads us to the most unusual aspect of this clinical case: the patient must have carried so many tuberculomas for long virtually asymptomatically. Although asymptomatic carriage of tuberculomas has been described in the literature [9,10], we could not identify any article describing such a high number of tuberculomas carried without any symptoms or signs whatsoever.

TBM has a high mortality. Case fatality ratios of 20-67% have been recorded even after adequate anti-TB medication, with patients with AIDS faring the poorest [11]. This problem with unfavorable outcomes persists in locations with higher resources, where mortality and neurologic sequelae rates can still reach 50%. One reason is the laboriousness and limited sensitivity of available diagnostic tools, which may result in diagnosis when CNS involvement is too advanced for effective treatment [12].

Mortality predictors for TBM have been extensively studied, but there is significant variation in findings. A study by Kumar et al. reports 15 studies with substantial variations of purported predictors [13]. The predictors that are repeated in more than one study include decreased Glasgow Coma Score, hydrocephalus, British Medical Research Council (BMRC) stage III, age over 40, duration of illness, seizures, history of TB, extraneural TB, infarctions, anemia, motor deficits or hemiparesis, low CSF glucose, low CSF/blood glucose ratio, and delay in treatment. These studies were done in different settings and populations, so these variable findings may not be generalizable worldwide.

However, how public health and policy shortcomings affect CNS-TB mortality has not been researched as much. Out of these issues, diagnostic and treatment delay predictors and outcomes have received greater focus. Some of the risk factors found for these delays include lack of symptom severity, limited awareness of TB, attribution of symptoms to a different cause, negative disposition towards medicine, and barriers to care [14]. Our patient had experienced several difficulties that may have predisposed him to CNS-TB, delayed diagnosis, or complicated the treatment, ultimately resulting in a lethal outcome. He was diagnosed with AIDS and toxoplasmosis in Ukraine, a country with a higher HIV incidence [15]. Upon return to Georgia, he didn't have any documentation or imaging, limiting his HIV history to word-of-mouth and rendering comparative imaging impossible. Also, having HIV and complications diagnosed elsewhere may have hindered his integration into Georgia's HIV programs, compromising his medical care and education regarding drug adherence.

Although Georgia has recently made significant progress in reducing the incidence of drug-resistant TB, it is still a work in progress given the complicated baseline of a decade ago [16]. The drug regimens used when primary drugs fail are considerably more toxic and less effective [17]. This issue might have caused this patient's severe side effects, such as hepatitis and, ultimately, failure of treatment. Although GeneXpert MTB/RIF can test for rifampicin sensitivity in less than two hours [18], susceptibility to other drugs is tested much slower. For example, resistance detection turnaround for the BACTEC^TM^ system (Becton, Dickinson and Company, Franklin Lakes, New Jersey, United States) is around three weeks, while culture methods can take almost twice as long [19]. Primarily, dedicated TB facilities test TB resistance in Georgia, so patients may be too advanced when transferred there from other hospitals for resistance detection to make any real clinical difference. This patient was rifampicin-sensitive, disqualifying him from having MDR-TB. Although sensitivity to other drugs was not tested, the combination treatment still failed to improve his symptoms, suggesting that he could have been resistant to other drugs used in the regimen, including INH, fluoroquinolones, or linezolid, the state that WHO calls "poly-resistant TB." This umbrella term collects various resistance patterns provided the bacteria is resistant to INH or rifampicin but not both [20].

Additionally, changing the treatment location from a general hospital to a dedicated TB facility and then back because the TB facility doesn't maintain an ICU may have also complicated the management of this patient whose life was hanging on a thread.

Considering the above facts, further research should address these public health and policy issues alongside more traditional clinical challenges. Our suggestions include studying which organizational and policy approaches can help detect CNS-TB better at the asymptomatic stage, emphasizing the challenges of fully integrating patients diagnosed with HIV abroad into national HIV programs and its effects on developing CNS-TB, creating maps of different resistance patterns grouped under the "poly-resistant TB" moniker and defining their prognoses regarding CNS-TB development and outcomes, laying out the policy for streamlining the collaboration between TB-dedicated and general hospitals and earlier checks for TB resistance that could improve the clinical course, and determining whether there is any difference between outcomes of patients treated in TB-dedicated facilities with vs. without ICU. Having answers to these questions may help optimize care for patients with this complicated diagnosis, which is often fatal despite medicine's best efforts.

## Conclusions

This clinical case emphasizes that a significant burden of CNS tuberculoma can be carried asymptomatically for extended periods while also demonstrating the risk of sudden clinical deterioration. It also sheds light on public health considerations that may predispose to CNS-TB or complicate its management. We recommend researching these issues further alongside the clinical variables of CNS-TB.
